# Gone to the Dogs—The Canon of Kitsch

**DOI:** 10.3201/eid3205.AC3205

**Published:** 2026-05

**Authors:** Lesli Mitchell

**Affiliations:** Centers for Disease Control and Prevention, Atlanta, Georgia, USA

**Keywords:** dogs, One Health, A Friend in Need, kitsch, art–science connection

**Figure:**
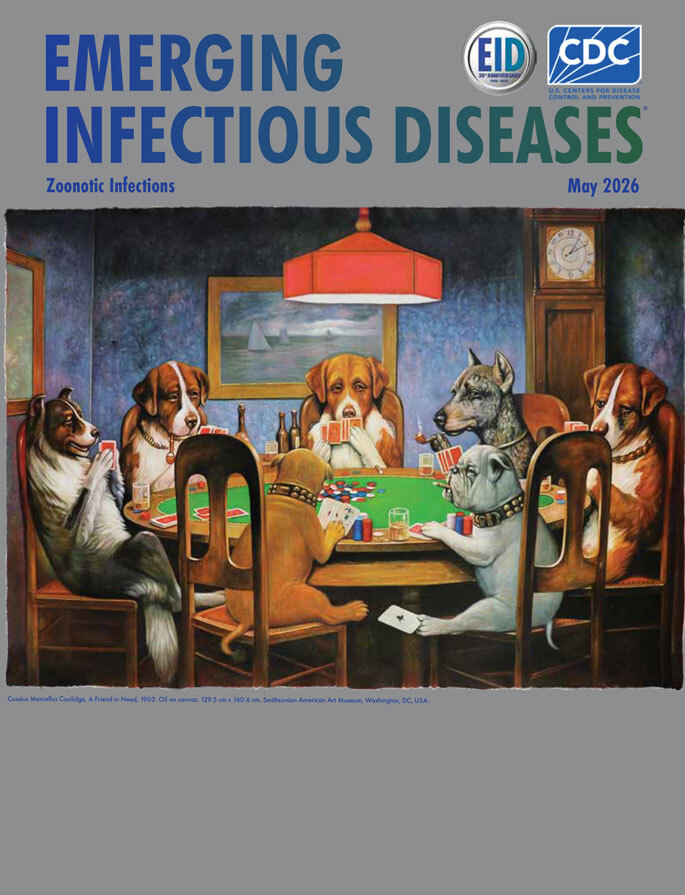
**Cassius Marcellus Coolidge, *A Friend in Need*, 1903.** Oil on canvas. 129.5 cm × 160.6 cm (51 in × 63.22 in). Smithsonian American Art Museum, Washington, DC, USA.

This issue’s cover art is a work of contradictions. It’s immediately recognizable, and yet it’s painted by “the most famous American artist you’ve never heard of.” In popular culture, it’s known as “Dogs Playing Poker,” but its actual title is *A Friend in Need*. Often referenced as a standalone work, in fact, it’s the fourth in a series of 16 paintings featuring dogs by artist Cassius Marcellus Coolidge (1844–1944). Although art critics do not consider the paintings fine art, Sotheby’s deigned to auction off one of Coolidge’s dogs playing poker paintings for an impressive $650,000 in 2015, and *A Friend in Need* currently resides on the wall of the Smithsonian American Art Museum in Washington, DC.

Since the painting’s wildly successful introduction in 1903, as an image in an advertising calendar, *A Friend in Need* has continued to be a part of the public imagination. The image is everywhere—in television, movies, music videos, hung as a poster in bars and man caves, even sold, still, as a calendar. The enduring popularity of the image has prompted art critics to explore the source of its lasting appeal. Art critics typically categorize the painting as kitsch, a term introduced in art in 1939 by influential New York art critic Clement Greenberg. In his essay “Avant-Garde and Kitsch,” published in *Partisan Review*, Greenberg defined kitsch as commercial art concerned with “magazine covers, illustrations, ads, slick and pulp fiction, comics, Tin Pan alley music, tap dancing, Hollywood movies.”

Coolidge’s work certainly fits that mold, perhaps even more so since the artist had no formal training. Cassius Marcellus Coolidge was born in New York in 1844 to abolitionist Quaker parents and was named after antislavery crusader Cassius Marcellus Clay. In his youth, he pursued a series of seemingly random jobs—druggist, sign painter, founder of a newspaper, even founder of a bank that still exists today—before settling into a career as an artist. He was hired by the advertising company Brown & Bigelow, which specialized in the production of advertising calendars, and it was there that Coolidge created 16 paintings of dogs known collectively as “Dogs Playing Poker.”

The series was an immediate hit: “These calendars proved to be massively successful, and Coolidge’s art found its way into millions of homes,” according to the blog The Automat. Part of the paintings’ success, both then and today, may be that despite Coolidge’s lack of professional training, his work ticked the boxes of established principles of composition. The composition is circular, anchored by the table in the center, with the objective of guiding the eye around the circle to each subject. The highly saturated green of the table is value-matched to the complementary strong red above it, in the hanging lamp. The background follows the rule of thirds, dividing the canvas, creating a triangle in the center, and drawing the viewer’s eye across the canvas in a diagonal line. In painting the background, Coolidge also makes use of a technique known as scumbling, scrubbing the brush onto the canvas to roughen it. He scumbles as he pairs that texture with the Doberman’s coat.

Coolidge’s composition also tells a human story, using the dogs as allegory, and upon close inspection the viewer is rewarded with a host of intriguing details. Many people enjoy the image without noticing the circumstances that give the painting its name, *A Friend in Need*. Subtly, in the foreground, we can see that the bulldog is cheating, passing an ace under the table to his companion. Once we see that, we see more: his companion has three aces in hand and needs only a fourth to win. The clock indicates the lateness of the hour, suggesting the game has been going on for a while. The bulldogs are clearly doing very well for themselves, with tall stacks of chips, compared with the pitiful scattering of chips held by the other dogs.

Coolidge also gives the dogs character and complexity. The St. Bernard in the middle eyes the Doberman suspiciously, and the Doberman in turn eyes the bulldog suspiciously. Or perhaps he’s looking beyond the bulldog to us, the viewers, asking, “Are you seeing this?” The St. Bernard on the right is lamenting his hand, knowing he’s going to lose again. The collie and St. Bernard on the left, who have a few more chips than the others, have half smiles, perhaps hopeful that this will be the hand that changes their fortunes.

For an artist with no formal training, Coolidge nevertheless knows how to engage his viewers and give them a rich and fun experience. In this accomplishment, the painting begins to escape the negative associations in Greenberg’s definition of kitsch, which he considers to be about money and ease: “Kitsch pretends to demand nothing of its customers except their money—not even their time.” Kitsch at its core is a manipulation, using sentiment to make money. Coolidge’s skill in both technique and subject expresses the playfulness of the scene without tipping the scale to sentimentality. The way in which Coolidge anthropomorphizes the dogs feels authentically affectionate. Even art critics respond to that. As Tamar Avishai says in his podcast, *The Lonely Palette*:

When I look at this painting, of course I think of the Cezanne card player paintings, but then the sort of art critic in me falls away and I just look at the dogs. I love dogs, and I think that everyone probably goes to the breed that appeals to them first or they have some life or childhood connection to.

The work encourages viewers to connect emotionally with the dogs. We are, after all, the only ones who know what the bulldogs are up to. We are invited to enjoy the fun of the scene, and we accept the invitation. In the same podcast, Avishai mentions his friend:

My buddy Wade has both PhD from MIT and a new Australian shepherd puppy, and he’s certainly not above wistfully commenting that if there had been an Aussie at the poker table, you know he would have cleaned up.

The genuine warmth that comes across in this work helps it transcend the derogatory commercial connotations of kitsch. Coolidge’s work has a stretchiness to it that accepts the classification as “The Mona Lisa of Kitsch” and at the same time evokes a fondness from those who give it that label. It’s a painting that occupies a unique position as an American cultural artifact, and we embrace it whether it hangs on the wall of a museum or a pool hall.

The enduring popularity of *A Friend in Need* also points to the enduring bond that humans have with their canine friends and other domesticated animals. The health of humans is closely connected to the health of animals and our shared environment. The expansion of the population into new areas, climate changes, and travel and trade have led to the spread of new or emerging zoonotic diseases. Approaches like One Health address such risks by promoting collaboration among experts worldwide in human, animal, and environmental health. The goal is to improve the health of both humans and animals, including livestock, wildlife, and the beloved pets that bring companionship, delight, and enrichment to our lives.
